# Xenon for the prevention of postoperative delirium in cardiac surgery: study protocol for a randomized controlled clinical trial

**DOI:** 10.1186/s13063-015-0987-4

**Published:** 2015-10-09

**Authors:** Layth Al Tmimi, Marc Van de Velde, Paul Herijgers, Bart Meyns, Geert Meyfroidt, Koen Milisen, Steffen Fieuws, Mark Coburn, Koen Poesen, Steffen Rex

**Affiliations:** Department of Anesthesiology, KU Leuven – University of Leuven, University Hospitals of Leuven, Herestraat 49, B-3000 Leuven, Belgium; Department of Cardiovascular Sciences, KU Leuven – University of Leuven, B-3000 Leuven, Belgium; Department of Cardiac Surgery, KU Leuven – University of Leuven, University Hospitals of Leuven, Herestraat 49, B-3000 Leuven, Belgium; Department of Intensive Care Medicine, KU Leuven – University of Leuven, University Hospitals Leuven, Herestraat 49, B-3000 Leuven, Belgium; Department of Intensive Care Medicine and Cellular and Molecular Medicine, KU Leuven – University of Leuven, Herestraat 49, B-3000 Leuven, Belgium; Department of Public Health and Primary Care, KU Leuven – University of Leuven, B-3000 Leuven, Belgium; I-Biostat, KU Leuven – University of Leuven, B-3000 Leuven, Belgium; Department of Anesthesiology, University Hospital of the RWTH Aachen, Aachen, Germany; Laboratory Medicine, KU Leuven – University of Leuven, University Hospitals Leuven, Herestraat 49, B-3000 Leuven, Belgium; Department of Neurosciences, Laboratory for Molecular Neurobiomarker Research, KU Leuven – University of Leuven, Herestraat 49, B-3000 Leuven, Belgium

**Keywords:** Xenon, Sevoflurane anesthesia, Postoperative delirium, Cardiac surgery

## Abstract

**Background:**

Postoperative delirium (POD) is a manifestation of acute postoperative brain dysfunction that is frequently observed after cardiac surgery. POD is associated with short-term complications such as an increase in mortality, morbidity, costs and length of stay, but can also have long-term sequelae, including persistent cognitive deficits, loss of independence, and increased mortality for up to 2 years. The noble gas xenon has been demonstrated in various models of neuronal injury to exhibit remarkable neuroprotective properties. We therefore hypothesize that xenon anesthesia reduces the incidence of POD in elderly patients undergoing cardiac surgery with the use of cardiopulmonary bypass.

**Methods/Design:**

One hundred and ninety patients, older than 65 years, and scheduled for elective cardiac surgery, will be enrolled in this prospective, randomized, controlled trial. Patients will be randomized to receive general anesthesia with either xenon or sevoflurane. Primary outcome parameter will be the incidence of POD in the first 5 postoperative days. The occurrence of POD will be assessed by trained research personnel, blinded to study group, with the validated 3-minute Diagnostic Confusion Assessment Method (3D-CAM) (on the intensive care unit in its version specifically adapted for the ICU), in addition to chart review and the results of delirium screening tools that will be performed by the bedside nurses). Secondary outcome parameters include duration and severity of POD, and postoperative cognitive function as assessed with the Mini-Mental State Examination.

**Discussion:**

Older patients undergoing cardiac surgery are at particular risk to develop POD. Xenon provides remarkable hemodynamic stability and has been suggested in preclinical studies to exhibit neuroprotective properties. The present trial will assess whether the promising profile of xenon can be translated into a better outcome in the geriatric population.

**Trial registration:**

EudraCT Identifier: 2014-005370-11 (13 May 2015).

## Background

Postoperative delirium (POD) is frequently observed after cardiac surgery with incidences ranging from 20 to 80 % [1, 2]. It is associated with short-term complications such as an increase in mortality, morbidity, costs and length of stay, but is also associated with long-term sequelae, including the development of persistent cognitive deficits, loss of independence, and increased mortality for up to 2 years [3].

The pathophysiology of POD is complex and thought to be related to cerebral ischemia-reperfusion injury, endothelial dysfunction [4], neuroinflammation [5], neurotransmitterimbalances [6, 7] and direct neurotoxic effects of anesthetics [8]. The individual baseline risk of a patient to develop POD is determined by a variety of predisposing factors that are not possible to be modified, including age, male gender, psychiatric illness, cognitive impairment (e.g. dementia) and atherosclerotic disease [2].

POD has been suggested to be precipitated by intraoperative and postoperative triggers. In contrast to the predisposing factors, precipitating factors are amenable to modification and have thus become a target of prevention strategies. Unfortunately, the unambiguous identification of precipitating factors for POD remains notoriously difficult. Numerous elements have been described, including (but not limited to) cardiovascular surgery, use of cardiopulmonary bypass (CPB), application of anesthetics and cognitively active medication, hypotension, decreased cardiac output, hypothermia and hypoxia [9, 10].

The noble gas xenon has been shown to offer neuroprotection in numerous in-vitro and in-vivo models of neuronal injury including models of post-cardiac surgery neurocognitive dysfunction [11, 12]. Neuroprotection by xenon is most likely achieved by diverse mechanisms including antagonism at the N-methyl-D-aspartate (NMDA) subtype of the glutamate receptor [13], an enhanced synthesis of prosurvival (B-cell lymphoma 2 and extra large) proteins and the suppression of neuronal apoptosis [14, 15]. Moreover, xenon preserves cerebral flow-metabolism coupling [16, 17] and possesses anti-inflammatory qualities that may directly interfere with the pathogenesis of POD [18]. Furthermore, the favorable physicochemical properties of xenon result in rapid clearance from the brain, thereby reducing any residual anesthetic effects that may predispose to POD [19, 20]. Last, xenon has been repeatedly demonstrated to cause less hemodynamic deterioration than conventionally used anesthetics [21–23]; therefore, it helps to avoid hemodynamic instability which is a precipitating factor for POD.

Therefore, we hypothesize that in older cardiac surgical patients, the use of xenon anesthesia significantly reduces the incidence of POD when compared to the conventionally used anesthetic sevoflurane. Additionally, the evaluation of outcome will include several secondary endpoints such as the duration and severity of POD, postoperative cognitive function as analyzed with the Mini-Mental State Examination (MMSE), length of intensive care unit (ICU) stay, hospital length of stay, the incidence of postoperative organ dysfunction and the incidence of (serious) adverse events ((S)AE).

## Methods/Design

### Study registration

The study will be performed according to the principles of the International Declaration of Helsinki and to the principles of good clinical practice (GCP) and is approved by the ethics committee of the University Hospitals Leuven, by the Clinical Trials Centre of the University Hospital Leuven, and by the “Federaal Agentschap voor Geneesmiddelen en Gezondheidsproducten.” The study is registered in the European Clinical Trials Database of the European Medicines Agency (EudraCT Identifier: 2014-005370-11, 13 May 2015). Any eventual changes of the study protocol will be reported to the ethics committee.

### Recruitment

Patients will be recruited by the principal investigator or co-investigator. Detailed information about the study background and protocol will be given, and any possible questions brought forward by the patients will be answered. Each eligible patient who is willing to participate in the study will sign a written informed consent before any particular study procedure.

### Study type

This prospective, randomized, controlled trial will be performed at the University Hospital of the KU Leuven by two types of investigators. Investigator I will assess the primary outcome (and the majority of the secondary outcome parameters) and is blinded to the group affiliation. Investigator II will perform anesthesia and cannot be blinded to the treatment groups due to the administration of the anesthetic via a dedicated anesthesia machine and the mandatory monitoring of anesthetic concentrations [24].

### Randomization

Patients will be randomized using a computer-generated permuted block randomization sequence (variable block-size, 1:1 allocation). Randomization will be stratified by the European System for Cardiac Operative Risk Evaluation (EuroSCORE) II (Stratum I: EuroSCORE ≤ 3; Stratum II: EuroSCORE > 3). A masked randomization procedure will be used in which group assignments are hidden in sealed, opaque, sequentially numbered envelopes that will only be opened upon arrival of the patient to the operation room (OR).

### Inclusion criteria

- Age > 65 years
- Patient scheduled for elective heart surgery with the use of CPB
- Able to read and understand the research materials

### Exclusion criteria

- Inability to give informed consent
- Intraoperative need for one-lung-ventilation and/or for an increased fraction of inspired oxygen (FiO_2_ > 50 %)
- Chronic obstructive pulmonary disease (COPD) GOLD > II
- Critical preoperative state (as defined by the requirement of inotropic support or intra-aortic balloon pump (IABP), ventricular tachycardia or ventricular fibrillation, preoperative cardiac massage, preoperative ventilation before anesthetic room, preoperative acute renal failure (anuria or oliguria < 10 ml hr^−1^) [25]
- Disabling neuropsychiatric diseases (dementia, schizophrenia, epilepsy or mental retardation)
- Presence of delirium at baseline as screened with the 3-minute Diagnostic Confusion Assessment Method (3D-CAM) [26]
- Alcohol abuse: (defined as a CAGE score ≥ 2) [27]
- History of drug abuse
- History of stroke or traumatic brain injury with residuals
- Increased intracranial pressure
- Hypersensitivity to the study medications
- Risk for malignant hyperthermia

### Anesthesia and intervention

**Fig. 1:**
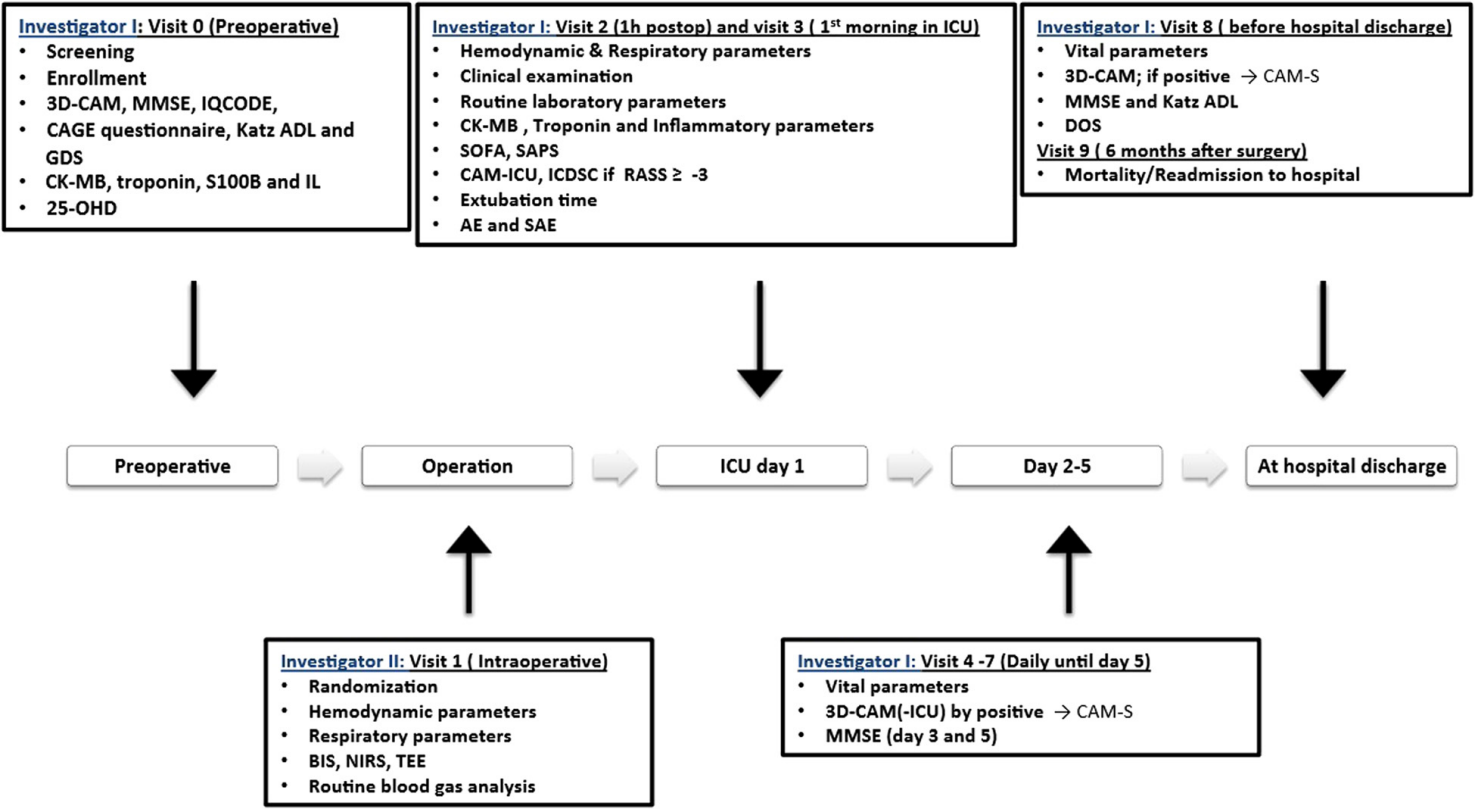
Schematic illustration of the study visits. 25-OHD, 25-hydroxy vitamin D; 3D-CAM, 3-minute Diagnostic Confusion Assessment Method; AE, adverse event; APACHE II, Acute Physiology and Chronic Health Evaluation II; BIS, bispectral index; CAM-S, Confusion Assessment Method Severity score; CCI, Charlson Comorbidity Index; DOS, Delirium Observation Scale; GDS, Geriatric Depression Scale; ICDSC, Intensive Care Delirium Screening Checklist; ICU, intensive care unit; IL, interleukin; IQCODE, Informant Questionnaire on Cognitive Decline in the Elderly; Katz ADL, Katz Index of Activities of Daily Living; MMSE, Mini-Mental State Examination; NIRS, near-infrared spectroscopy; RASS, Richmond Agitation-Sedation Scale; SAPS, Simplified Acute Physiology Score; SOFA, Sequential Organ Failure Assessment score; SAE, serious adverse events; TEE, transesophageal echocardiography

The different steps of the study visits are summarized in Fig. 1.

#### Baseline assessment (Investigator I)

Investigator I will obtain written informed consent and record baseline data (demographic data, medical and surgical history, routine clinical examination, Charlson comorbidity index (CCI) [28], results of preoperative chest X-ray, electrocardiogram (ECG) analysis, echocardiography and standard laboratory parameters). In addition, the MMSE [29], the 3D-CAM, the Geriatric Depression Scale (GDS) [30] and an interview with a family member using the short form of the Informant Questionnaire on Cognitive Decline in the Elderly (IQCODE) [31] will be performed the day before surgery. Patients with alcohol abuse will be identified with the CAGE questionnaire [27] and excluded. Besides, patients’ preoperative functional status will be assessed using the Katz Index of Activities of Daily Living (ADL) [32].

#### General anesthesia

Patients must be in a fasting state for 6 hours prior to anesthesia. Patients will be premedicated with lorazepam per os 0.03 mg kg^−1^ 1 hour before surgery (patients > 80 years with lorazepam 0.015 mg kg^−1^).

After 5 minutes of pre-oxygenation (FiO_2_ = 1.0), general anesthesia will be induced with a combination of remifentanil 0.5 μg kg^−1^ min^−1^ followed by a bolus of intravenous propofol 0.5–1 mg kg^−1^. Tracheal intubation will be facilitated by the bolus administration of cisatracurium (0.2 mg kg^−1^). Afterwards, the sealed randomization envelopes will be opened and patients will be randomly allocated to the two treatment groups in which general anesthesia will be maintained with either:

- xenon 40–60 % in oxygen (FiO_2_ = 0.5–0.6) (xenon-group), or
- sevoflurane 1.0–1.4 % (FiO_2_ = 0.5–0.6) (sevoflurane-group)

In both groups, anesthetic concentrations will be titrated according to the clinical observation of vegetative signs for the depth of anesthesia (including heart rate (HR), blood pressure, sweating, etc.) and to the instantaneously registered electroencephalogram (EEG)-monitoring in order to reach a bispectral index (BIS) value between 40 and 60. Cisatracurium will be administered at the induction of anesthesia. Further curarization will be left upon the discretion of the attending anesthesiologist.

Analgesia will be achieved with a continuous infusion of remifentanil (0.15–0.25 μg kg^−1^ h^−1^), starting after induction of anesthesia. The dose of remifentanil will be adjusted by increments of 0.05 μg kg^−1^ min^−1^ when the patient develops sweating, shows spontaneous movements and/or a sudden increase in HR or arterial pressure by more than 20 % [33]. *During the CPB period*, the investigational treatment will be discontinued in both groups and replaced with a target-controlled infusion (TCI) of propofol with calculated plasma concentrations of 1–2 μg ml^−1^ that will be titrated according to the BIS-monitoring. After completion of surgery, the interventional treatment will be stopped, and a bolus of intravenous (IV) piritramide 0.2 mg kg^−1^ will be given. Patients will be transferred to the ICU under mono-sedation with propofol.

### Monitoring and hemodynamic management

Hemodynamic and respiratory monitoring will be provided according to our institutional routine including the placement of an arterial, a central venous and a pulmonary artery catheter. Whenever available, the following parameters will be recorded every 15 minutes from the pre-anesthetic period to the end of surgery: peripheral oxygen saturation (SpO_2_), invasive arterial blood pressure (IBP), HR, central venous pressure (CVP), cardiac output (CO), mixed venous oxygen saturation (SvO_2_), mean pulmonary artery pressure (MPAP), fraction of inspired oxygen (FiO_2_), end-tidal carbon dioxide (etCO_2_), body temperature, BIS, urine output, blood loss. Additionally, cerebral tissue oxygenation (rSO_2_) will be assessed by near-infrared spectroscopy (NIRS). Besides, transesophageal echocardiography (TEE) will be performed during anesthesia according to our institutional standards. Fixed measurements will be achieved for blood gas and a complete hemodynamic profile at the following time points: T0 = prior to induction, T1 = after induction, T2 = after sternotomy, T3 = after weaning from CPB, T4 = after sternal closure and T5 = end of surgery.

Intraoperative hemodynamic management will be standardized according to our clinical routine. Basic fluid substitution will be performed with 1 ml kg^−1^ h^−1^ balanced crystalloid solution. Hemodynamic stability will be defined as cardiac index > 2.5 L min^−1^ m^−2^, SvO_2_ > 70 % and mean arterial blood pressure of > 65 mmHg (between 50 and 60 mmHg during CPB). In case of hypovolemia (as detected by TEE or visual inspection of the heart), colloid solutions (gelatin) will be infused. Packed red blood cells (PC) will be transfused when the hemoglobin content is less than 8.0 g dL^−1^. A norepinephrine infusion will be administered when hemodynamic stabilization cannot be achieved despite adequate volume loading. The administration of inotropes, vasodilators, PC, fresh frozen plasma (FFP) and platelets will be left at the discretion of the attending anesthesiologist.

### Cardiopulmonary bypass

CPB will be performed in mild to moderate hypothermia on a conventional CPB circuit with cardiac arrest induced by antegrade or retrograde infusion of cold crystalloid cardioplegic solution. Extracorporeal circulation will be performed with a pump flow of 2.2–2.5 L min^−1^ m^−2^. Prior to CPB, 300–350 IU kg^−1^ heparin will be administered to achieve an activated clotting time of > 400 s. After weaning from CPB, heparin will be antagonized with protamine in a ratio of 1:1. Furthermore, propofol TCI will be stopped and the investigational treatment will be re-administered.

### Intensive care unit treatment

On the ICU, analgesia will be achieved with a continuous infusion of piritramide, supplemented by systemic acetaminophen (1–2 g, administered IV every 8 hours) during the first postoperative day. Standard tracheal extubation and patients discharge criteria will be applied.

As part of standard care, a bundle of non-pharmacological interventions will be applied to prevent delirium, including:

- Active detection and treatment of pain
- Early mobilization
- Removal of catheters, drains, probes, etc. as soon as possible
- Timely recognition and treatment of medical problems such as stroke, infections, electrolyte imbalances, hypoxia, heart failure, hypotension, dehydration, urinary retention, etc.
- Strict avoidance of medications known to trigger POD (in particular, benzodiazepines and steroids)

Whenever a POD occurs, pharmacological treatment based on our institutional standards of care, will be intiated.

Once standard discharge criteria will be met, the patients will be transferred to the ward.

### Study outcomes

#### Primary endpoint

Incidence of postoperative delirium as assessed by 3D-CAM and/or CAM-ICU.

The primary study endpoint will be the incidence of POD during the first 5 postoperative days. Hence, the null hypothesis states that there is no difference between both groups in POD incidence (yes/no) during the first 5 postoperative days. Through ICU-stay, patients will be assessed daily for the presence of POD by trained research nurses (blinded to the group assignment) using the CAM adapted for the ICU (CAM-ICU) [34]. In addition, we will perform a daily chart review for the results of the Intensive Care Delirium Screening Checklist (ICDSC) [35] over the previous 24 hours, which is a standard assessment, conducted during each 8-hour shift by the bedside ICU nurse. Patients with a Richmond Agitation-Sedation Scale of < −3 will be considered unconscious and not evaluable for POD [36]. After transferal to the ward, patients will be assessed by trained research personnel on a daily basis for the presence of POD using the 3D-CAM. In addition, we will perform a daily chart review for the results of the Delirium Observation Scale (DOS) [37] over the previous 24 hours that will be conducted by the nurses. On both the ICU and the ward, the patients’ records over the previous 24-hour period will be checked to identify key words suggestive of POD (e.g. confused, agitated, aggressive, disorientated, drowsiness and delirious) and the administration of antipsychotic therapy [38, 39].

#### Secondary endpoints

Secondary endpoints include the duration of POD (total number of days and percentage of patients with duration of POD longer than 2 days; for patients who have POD on day 5, daily clinical assessments are continued until the symptoms resolve or until the patient is discharged), the severity of POD (assessed with the delirium severity measure based on CAM (CAM-S) [40], use of physical restraints, postoperative cognitive function as analyzed with the MMSE at postoperative day 3, day 5, the day before discharge and at 6 months post surgery, and preoperative and postoperative ADL functioning as analyzed with the Katz ADL scale at baseline and the day before discharge and at 6 months post surgery.

In addition, the administration of anti-delirium medications (such as haloperidol, anxiolytics, dexmedetomidine, etc.) and duration of sedation will be registered and compared between both groups.

Other secondary endpoints will be:

- Incidence of MACCE (major adverse cardiac and cerebral events, occurring until discharge from hospital and in the first 6 postoperative months); i.e. death from any cause; perioperative myocardial infarction, requirement of surgical revisions at the coronary arteries; postoperative coronary angioplasty; and stroke. Myocardial infarction is defined as the occurrence of a new Q wave in addition to a rise of more than 10 ng ml^−1^ of troponin in the early postoperative period or any episode of chest pain with typical rise and fall of cardiac enzymes thereafter
- Other cerebrovascular accidents not included in MACCE (transient ischemic attacks, reversible ischemic neurologic deficit)
- Postoperative renal function (as assessed by serum creatinine and blood urea nitrogen (BUN) levels)
- Requirement for blood (product) transfusion
- Incidence of intraoperative awareness as detected by the use of a structured Brice questionnaire [41] that will be performed one day after ICU discharge
- Duration of postoperative ICU-stay and hospital stay
- Severity of postoperative critical illness as indicated by the Acute Physiology and Chronic Health Evaluation (APACHE) [42] II score once within 24 hours after admission to the ICU and the Sequential Organ Failure Assessment (SOFA) score [43]
- Incidence of serious adverse events (SAE) and suspected unexpected serious adverse reactions not included above. According to the International Conference on Harmonization of Technical Requirements for Registration of Pharmaceuticals for Human Use (ICH), an adverse event (AE) will be defined as “any untoward medical occurrence in a patient or clinical investigation subject administered a pharmaceutical product and which does not necessarily have to have a causal relationship with this treatment.” A SAE is “any AE that is life threatening – results in death – requires patients re-hospitalization and/or prolongation of existing hospitalization – or results in patient’s disability” [44]
- Intraoperative hemodynamic and respiratory profile
- Intraoperative use of inotropic/vasoactive medication

### Blood samples and laboratory parameters

Because of a possible correlation between preoperative vitamin D levels and the occurrence of POD, serum-levels of 25-hydroxy vitamin D (25-OHD) will be checked for all patients preoperatively [45, 46]. Furthermore, different blood samples will be collected at baseline (visit 0), at 1 hour after end of surgery (visit 2) and postoperative day 1 (visit 3). From these samples, the following parameters will be examined:

- Markers of glial injury: serum protein S100β
- Hemoglobin and hematocrit
- Cardiac markers: CK-MB and troponin T-hs
- C-reactive protein (CRP), leukocyte counts, serum cytokines (interleukin (IL)-6, IL-10)
- Respiratory markers: SaO_2_, PaO_2_ PaCO_2_, arterial pH, bicarbonate and base excess
- Renal markers: serum creatinine and blood urea nitrogen
- Hepatic markers: serum alanine aminotransferase (ALT), aspartate aminotransferase (AST), gamma-glutamyl transferase (GGT) and total bilirubin
- Serum electrolytes and serum blood sugar concentration

Additional blood samples may be collected, at the investigator’s or other physician’s judgment, in case of technical problems during the initial assay performed or missing baseline values prior to surgery and/or clinical abnormalities necessitating specific additional measurements.

### Statistical analysis and sample size calculation

All statistical analyses will be performed using a commercially available software package (SAS software, version 9.2 of the SAS System for Windows, SAS Institute Inc., Cary, NC, USA). All tests will be 2-sided, and a *p* < 0.05 will be considered to indicate statistical significance.

For the primary outcome, the incidence of POD, a logistic regression adjusting for the stratification variable EuroSCORE will be used to compare both groups. Sensitivity analyses will be performed to evaluate the impact of the non-evaluable patients (expected to be less than 5 %: e.g. deceased patients, transferred to other centers, extremely sedated patients) during the 5 postoperative days. First, a “worst case scenario” will be considered assuming absence of POD for all non-evaluable patients in the conventional anesthesia group and presence of POD for all non-evaluable patients in the xenon-anesthesia group. Second, a multiple imputation approach will be used imputing a POD value for the non-evaluable patients, with as imputation model a logistic regression fitted in both groups separately [47] with baseline characteristics as categorical and continuous predictors. Twenty imputed datasets will be created and results will be averaged appropriately.

For the secondary outcomes, the used statistical test will depend on the type and distribution of the considered outcome. Normally distributed data (verified with the Shapiro-Wilk *W* test statistic) will be compared using an analysis of covariance (ANCOVA) with the dichotomized EuroSCORE as binary covariate. Intergroup comparisons of non-normally distributed data will be performed using the stratified version of the Mann–Whitney *U* test. Differences in proportions will be analyzed using the exact test for a stratified 2x2 table. Time-to-events (occurrence of POD) will be assessed from end of surgery until the occurrence of the POD. Patients will be censored if they do not experience POD at the time of the last follow-up. To obtain the cumulative distribution curves for the POD times, Kaplan-Meier estimates will be used and groups will be compared using the stratified log-rank test. A linear model for longitudinal measurements with an unstructured covariance matrix for the fixed points in time (i.e. a multivariate regression model using a direct likelihood approach) [48] and the dichotomized EuroSCORE as a binary covariate will be used to evaluate the evolution of laboratory markers.

The study will be powered to detect the differences in primary outcome between the sevoflurane-group and the xenon-group. Based on recent observations from our institute [49], the incidence of POD after cardiac surgery with sevoflurane-anesthesia is approximately 40 %. A comparable incidence has already been reported earlier by our group [50] and has also been observed by other investigators [3]. Using a 2-sided test for the detection of differences between proportions (with alpha = 5 % and applying a continuity correction), at least 91 patients per group (182 in total) are required to show with 80 % power a 50 % reduction in the incidence of POD. To compensate for possible drop-outs, we will include 190 patients in total. They will be randomized into both groups in a 1:1 ratio.

Since the needed sample size depends extremely on the assumed POD rate, a sample size recalculation (SSR) will be performed after inclusion of 100 patients. This SSR will be based on a blinded evaluation of the overall POD rate [51]. Since the SSR is fully blinded (i.e. the group allocation is not known), this analysis can be performed by a member of the study steering committee. Applying the assumed relative risk (relative reduction of the POD by 50 %) on the overall POD rate, the power will be recalculated. For example, suppose that the overall POD rate after inclusion of 50 subjects in each group equals 24 %, instead of the expected 30 % (40 % and 20 %, respectively). Then, based on the assumed 50 % relative reduction, this corresponds to 16 % and 32 % for xenon and sevoflurane, respectively, which would require 123 patients in each group instead of 91 patients to maintain 80 % power. Based on this information, the study steering committee will decide on extending the accrual time. Note that based on the result of the SSR, the planned sample size will only be maintained or increased (not lowered).

## Discussion

The primary aim of the current trial is to test whether general anesthesia with xenon significantly reduces the incidence of POD in older patients undergoing elective cardiac surgery with the use of CPB.

### Novelty

Numerous in-vitro and in-vivo studies have shown that the noble gas xenon owns neuroprotective properties [11, 52, 53]. Evidence on xenon-mediated neuroprotection in humans is, however, scarce. To the best of our knowledge, four randomized controlled trials tested the efficacy of xenon for the prevention of postoperative cognitive dysfunction (POCD). Unfortunately, these studies produced controversial results [19, 20, 54, 55], most probably owing to the inclusion of heterogenous patient populations, the diversity in types of surgery, different follow-up periods in which patients were assessed for POCD, the use of different test batteries, and – at least in part – inadequate sample sizes. Moreover, not all studies focused on patients known to exhibit a particularly high risk for the development of neurocognitive complications.

Our clinical trial will assess the efficacy of xenon for the prevention of POD in cardiac surgery. Due to its scarcity, xenon is extremely costly. These expenses will only be justified when the promising observations with xenon in preclinical experiments can be translated into a better outcome for the geriatric population. POD has been demonstrated to be associated with important short-term and long-term complications and occurs particularly frequently in patients older than 65 years. Our study will, therefore, focus on an extremely vulnerable population. We will employ well-validated methods for delirium screening and also assess long-term neurocognitive function.

### Limitations

Investigator II, who performs the xenon or sevoflurane anesthesia, cannot be blinded to the kind of intervention. However, this investigator will have to adhere to a strict hemodynamic treatment protocol in order to ensure equivalent management in both groups and avoid bias. Moreover, investigators blinded to the group affiliation will assess all postoperative outcomes of the current trial, including the primary endpoint and the majority of secondary outcomes.

Furthermore, the noble gas xenon will only be administered during the intraoperative period. This is exactly the time period in which neuronal injury leading to POD and neurocognitive deterioration is expected to occur and during which neuroprotective interventions should presumably have the greatest benefit. However, any potential advantages of xenon with respect to recovery characteristics could be masked by postoperative events or interventions in the ICU or the nursing ward.

### Benefits for the participating patients

There is no guarantee that the use of xenon, instead of standard treatment with sevoflurane alone, will provide any medical advantage to the participant.

### Safety issues

The interventional treatment will be administered to patients under advanced hemodynamic monitoring in the setting of a fully equipped cardiac surgical operation room. This enables immediate detection and treatment of adverse events. Xenon inhalation will be immediately stopped in case that the study patient will show a life-threatening deterioration. After leaving the operation room, all patients will be closely monitored by the study team for the occurrence of eventual (S)AEs during the whole postoperative period until hospital discharge. Moreover, the inclusion of each individual patient into the study is indicated in the electronic hospital information system and hence visible to all physicians and nurses involved in the care of this patient. This will facilitate reporting of (S)AEs to the principal investigator. Finally, suspected unexpected serious adverse reactions will be reported by the principal investigator to the federal health authorities.

## Trial status

The enrollment period is planned over a 36-month period starting in September 2015. The end of patient enrollment is expected for October 2018. A subsequent period of 6 months is planned for the primary data evaluation and statistical analysis including the 6-month follow-up for the assessment of long-term outcome. An additional period of 3 months is planned for remaining data evaluation, statistical analysis and publication of the results.

## Abbreviations

25-OHD: 25-hydroxy vitamin D
3D-CAM: 3-minute Diagnostic Confusion Assessment Method
ADL: Katz Index of Activities of Daily Living
AE: adverse events
ALT: alanine aminotransferase
ANCOVA: analysis of covariance
APACHE II: Acute Physiology And Chronic Health Evaluation II
AST: aspartate aminotransferase
BIS: bispectral index
BUN: blood urea nitrogen
CAM: Confusion Assessment Method
CAM-S: Confusion Assessment Method Severity score
CAM-ICU: CAM adapted for the ICU
CCI: Charlson comorbidity index
COPD: chronic obstructive pulmonary disease
CO: cardiac output
CRP: C-reactive protein
CPB: cardiopulmonary bypass
CVP: central venous pressure
DOS: Delirium Observation Scale
ECG: electrocardiogram
EEG: electroencephalogram
etCO_2_: end-tidal carbon dioxide
FFP: fresh frozen plasma
FiO_2_: fraction of inspired oxygen
FWO: Research Foundation Flanders
GCP: good clinical practice
GDS: Geriatric Depression Scale
GGT: gamma-glutamyl transferase
HR: heart rate
IABP: intra-aortic balloon pump
IBP: invasive blood pressure
ICH: Technical Requirements for Registration of Pharmaceuticals for Human Use
ICDSC: Intensive Care Delirium Screening Checklist
ICU: intensive care unit
IL: interleukin
IQCODE: Informant Questionnaire on Cognitive Decline in the Elderly
IV: intravenous
MACCE: major adverse cardiac and cerebral events
MMSE: Mini-Mental State Examination
MPAP: mean pulmonary artery pressure
NMDA: N-methyl-D-aspartate
NIBP: non-invasive blood pressure
NIRS: near-infrared spectroscopy
OR: operation room
PaCO_2_: partial pressure of carbon dioxide in the blood
PC: packed cells
POCD: postoperative cognitive dysfunction
POD: postoperative delirium
rSO_2_: regional cerebral tissue oxygenation
RASS: Richmond Agitation-Sedation Scale
SaO_2_: arterial oxygen saturation
SAPS: Simplified Acute Physiology Score
SOFA: Sequential Organ Failure Assessment score
SAE: serious adverse events
SpO_2_: peripheral oxygen saturation
SSR: sample size recalculation
SvO_2_: mixed venous oxygen saturation
TCI: target-controlled infusion
TEE: transesophageal echocardiography
